# Metabolic risk factors in young adults infected with HIV since childhood compared with the general population

**DOI:** 10.1371/journal.pone.0206745

**Published:** 2018-11-08

**Authors:** Elise Arrive, Jean-Paul Viard, Benoît Salanave, Catherine Dollfus, Sophie Matheron, Véronique Reliquet, Elisa Arezes, Laura Nailler, Corinne Vigouroux, Josiane Warszawski, on behalf of the ANRS CO19 COVERTE and ENNS study groups

**Affiliations:** 1 Inserm, Center for Research in Epidemiology and Population Health, Paris, France; 2 Unité de Formation et de Recherche d’Odontologie, Université de Bordeaux, France; 3 Centre Hospitalier Universitaire de Bordeaux, France; 4 Centre de Diagnostic et de Thérapeutique, Hôtel-Dieu, Assistance Publique-Hôpitaux de Paris, Paris, France; 5 Unité de Recherche EA 7327, Faculté de Médecine Paris Descartes, Paris, France; 6 Equipe de Surveillance et d’Epidémiologie Nutritionnelle (ESEN), Santé publique France, Université Paris-13, Centre de recherche en épidémiologie et statistiques COMUE Sorbonne Paris Cité, Bobigny, France; 7 Pediatric Hemato-Oncology,Hopital Trousseau, Assistance Publique-Hôpitaux de Paris, Paris, France; 8 Hopital Bichat-Claude Bernard, Assistance Publique-Hôpitaux de Paris, Paris, France; 9 Unité Mixte de Recherche 1137, INSERM, Université Paris 7, Paris, France; 10 Department of Infectious Diseases and CIC UIC 1413 INSERM, Centre Hospitalier Universitaire de Nantes, Nantes, France; 11 Sorbonne Université, Inserm Unité Mixte de RechercheS 938, Saint-Antoine Research Centre, Institute of Cardiometabolism and Nutrition (ICAN), Paris, France; 12 Assistance Publique-Hôpitaux de Paris, Saint-Antoine Hospital, Biology and Molecular Genetics and Endocrinology Departments, National Reference Center for Rare Diseases of Insulin Secretion and Insulin Sensitivity, Paris, France; 13 Université Paris-Sud, Le Kremlin-Bicêtre, France; 14 Assistance Publique-Hôpitaux de Paris, Hôpital Bicêtre, Le Kremlin-Bicêtre, France; Tulane University School of Public Health and Tropical Medicine, UNITED STATES

## Abstract

**Aim:**

Metabolic risk factors are poorly documented for the first generation of young adults who have lived with HIV since childhood. We compared their metabolic profile with that of adults of same age from the general population.

**Methods:**

We conducted a cross-sectional analysis of data from two populations: (1) COVERTE (ANRS-CO19), a French national cohort of 18 to 30-year-old patients HIV-infected since childhood, and (2) ENNS, a national cross-sectional population-based household survey on nutrition. Body mass index (BMI), blood pressure, waist circumference, fasting glucose, triglycerides, and HDL-, LDL- and total cholesterol were measured in both studies. Direct standardization on overweight and education level and logistic regression were used to compare the prevalence of metabolic abnormalities between the two populations.

**Results:**

Data from 268 patients from COVERTE and 245 subjects from ENNS were analyzed. Tobacco use was similar in both groups. HIV-infected patients had increased mean waist-to-hip ratio and triglycerides to HDL-cholesterol ratio and decreased mean HDL-cholesterol as compared to their counterparts from the general population in both genders. In HIV-infected patients, metabolic syndrome was identified in 13.2% of men (95% confidence interval [CI]: 7.1–19.2) and 10.4% (95% CI: 5.4–15.3) of women versus 10.6% (95%CI: 1.5–19.7) and 1.7% (95%CI: 0–4.1) in subjects from the general population, respectively.

**Conclusion:**

Young adults infected with HIV since childhood had a higher prevalence of dyslipidemia and metabolically detrimental fat distribution than adults of same age of the general population, supporting close monitoring for cardiometabolic diseases.

## Introduction

Cardiovascular diseases are an important cause of non-AIDS-related morbidity and mortality in HIV-infected patients. They are the third cause of death of HIV-infected patients after AIDS events and hepatic diseases [1]. In a previously studied French cohort, the age- and sex- standardized risk of myocardial infarction in HIV-infected patients relative to the general population was 1.5 [95% confidence interval (CI) 1.3–1.7] overall, 1.4 (95% CI 1.3–1.6) in men, and 2.7 (95% CI 1.8–3.9) in women [2]. Myocardial infarctions occur at a younger age in HIV-infected patients, suggesting premature aging of the cardiovascular system [3]. Multiple synergistic risk factors, either traditional (tobacco use, components of the metabolic syndrome, age) or HIV-related (exposure to antiretroviral therapy [ART], immune/inflammatory alterations, lipodystrophy syndrome, decreased insulin sensitivity), may contribute to accelerated atherosclerosis in HIV-infected patients [4, 5]. The prevalence of diabetes in European HIV-infected patients has approached that of the general population in recent years, including in France [6–8], but dyslipidemia and insulin resistance associated with ART remain significant risk factors [9]. HIV-infected adults (aged 35–44 years) treated with a protease inhibitor-based ART regimen had lower mean high-density lipoprotein (HDL) cholesterol levels, higher mean waist-to-hip ratio, and higher mean triglyceride levels than persons of the same age from the French general population [10]. They also exhibited a higher prevalence of smoking with an overall greater predicted risk of coronary heart disease.

Few studies have examined the cardiovascular risks in the first generation of young adults living with HIV since birth or childhood. This is an emerging population, due to the improved prognosis of HIV infection treated with powerful ART. The cardiometabolic risk of HIV-infected patients has significantly declined over the last few years, due to broadened treatment indications, use of less toxic antiretrovirals, and better monitoring [11], but cardiometabolic features in young adults HIV-infected during childhood may differ from those in individuals infected during adulthood, for several reasons. These include the acquisition of HIV at a young age, during a period of physiological immaturity and of a strong capacity of immune regeneration through thymic activity; long exposure to HIV and long ART histories, with the use of first generation drugs associated with the highest metabolic toxicity; and conversely, the lack of exposure to behavioral risk factors, mainly tobacco use, during the first years of infection. In addition, fetal exposure to ART could increase the risk of cardiomyopathy [12].

A study conducted in the United States reported that coronary artery vessel wall thickness was significantly higher in young adults aged 15–29 years who acquired HIV early in life than in sex- and race-matched uninfected controls, indicating that this population is likely to present subclinical coronary vascular disease [13]. In addition, several studies conducted in teenagers and young adults infected with HIV early in life (mostly through mother-to-child transmission) showed a high prevalence of metabolic abnormalities, particularly dyslipidemia and insulin resistance [14, 15, 16], as well as coronary artery abnormalities [17].^.^ However, these studies were of small sample size and/or did not include non HIV-infected control groups.

The French national cohort of young adults living with HIV since childhood was designed to study their living conditions and long-term prognosis. The availability of a national population-based survey on nutrition provided the opportunity to compare their metabolic profile to that of the adults of the same age living in France.

## Patients and methods

### Data sources

The ANRS COVERTE-CO19 (S1 and S2 Files) is an ongoing prospective cohort of adults diagnosed with HIV infection before 13 years of age (verified to be perinatally-acquired for almost all patients), aged 18 to 25 years at inclusion, and enrolled in 88 sites (77 in metropolitan France, six in French overseas cities, and five in Brussels and Liège, Belgium). One third of the patients has been followed prospectively in the national cohort of HIV-infected children (Enquête Périnatale Française, ANRS CO1) since birth. For the ANRS COVERTE-CO19 cohort, an annual standardized questionnaire is completed by the medical team to collect clinical, therapeutic, and biological parameters from routine follow-up every six months, including fasting glucose, triglycerides, and HDL-, LDL-, and total cholesterol; additional details on living conditions and behaviors are collected through a self-administered questionnaire completed during each visit. Enrolment started in June 2010.

The ENNS (Etude Nationale Nutrition Santé, S3 and S4 Files), a French national cross-sectional population-based household survey, was conducted from February 2006 to July 2007. The main objective was to describe nutritional status in metropolitan France. It was based on a sampling design that provided a large representative sample of the population living in France (excluding the island of Corsica and French overseas territories) [18]. The ENNS design consisted of three-stage probability sampling with unequal inclusion probabilities stratified by region and degree of urbanization. A subgroup of voluntary participants had clinical examinations and provided biological samples. Post-stratification was performed to adjust for gender, the national census data on age, level of education, presence of at least one child in the household, season of medical examination, and the availability of weight, height, and total cholesterol measures. Data were collected in medical examination centers of the French National Health Insurance System (Caisse Nationale d’Assurance Maladie des travailleurs salariés) or at home.

COVERTE and ENNS received the approval of the Ethics Committee of the Ile de France III (Hôpital Cochin n^o^2264 and n°2738, respectively). Participants in both studies provided written informed consent.

### Study population

The study included all participants from both studies, aged 18 to 30 years, living in France, and not pregnant, except when information on the level of education, weight, height, or total cholesterol were missing. For HIV-infected patients, we selected individuals for whom completed self-questionnaire was available and biological parameters were collected within three months from the latest visit.

### Variables studied

#### Metabolic outcomes

The body mass index (BMI) was calculated as the ratio of the weight to the square of the height. Being overweight was defined as having a BMI ≥25. Metabolic parameters were measured using standard procedures. The metabolic abnormalities studied were those included in the Joint Interim Statement definition of metabolic syndrome [19]: blood pressure ≥130/85 mmHg or the use of blood pressure-lowering medication; waist circumference ≥94 cm (men) or ≥ 80 cm (women); fasting glucose ≥5.6 mmol/l or the use of antidiabetic medication; triglycerides ≥1.7 mmol/l or the use of lipid-lowering medication; HDL-cholesterol <1.03 mmol/l (men) or <1.29 mmol/l (women) or the use of specific medication. Metabolic syndrome was defined as having any three or more of the above metabolic abnormalities. We also considered the thresholds of LDL-cholesterol ≥ 3.4 mmol/L and total cholesterol ≥5.16 mmol/L [20].

#### Main exposure

The main exposure was the HIV status: HIV-infected since childhood (patients enrolled in COVERTE) or not HIV-infected (this was assumed, although systematic HIV testing was not performed, subjects enrolled in ENNS representing the general population living in France, without any subject having being exposed to ART).

The socio-demographic and behavioral characteristics collected in both studies were: gender, age (18–24 vs 25–30 years), marital status (married or living with a partner vs. being single), country of birth (metropolitan France vs. outside), employment status (working vs. not working or student), education level (no qualification or middle school vs. high school diploma or higher), and tobacco consumption (active vs. none or past), hormonal contraception, and psychotropic drug use.

The immuno-virological status and ART of HIV-infected patients were described by the level of CD4 cell counts and viral load, the CDC clinical stage, the type of ART at study visit, and the duration of exposure to any antiretroviral drug, including those reported to have metabolic toxicity (zidovudine, stavudine, didanosine, protease inhibitors).

### Statistical analysis

The analysis was conducted separately for men and women [21] on the database (S1 Dataset). The mean values of metabolic parameters and the prevalence of abnormalities were estimated with their 95% confidence intervals, according to the sampling design for ENNS. Crude numbers were used for both studies, but the percentages and means in the ENNS were weighted to take into account unequal probabilities of inclusion and post-stratification. Moreover, estimates in HIV-infected patients were standardized to adjust for the distribution of BMI (< 25 or ≥ 25) and the education level (under or at least a high school diploma) of the 18 to 30-year-old general population estimated from the ENNS survey, as these factors are strongly associated with metabolic status [21–23].

For HIV-specific parameters in COVERTE, the data for men and women were compared using Pearson Chi-Square or Fischer exact tests. Quantitative variables were compared using Kruskal-Wallis tests.

The association of the HIV status (as main exposure) with each metabolic parameter (as dependent variable) was studied using linear regression models for continuous parameters and logistic regression models for qualitative parameters. The models were systematically adjusted for being overweight and education level in both genders. Further adjustment for the use of oral contraceptives was performed in women as they are known to modify lipid metabolism [24].

A sensitivity analysis was conducted by excluding participants born outside France.

Statistical analyses were performed using the survey procedure of SAS 9.4. The threshold for statistical significance was 0.05. No adjustment was used for multiple testing.

## Results

### Description of the study population

**Fig 1:**
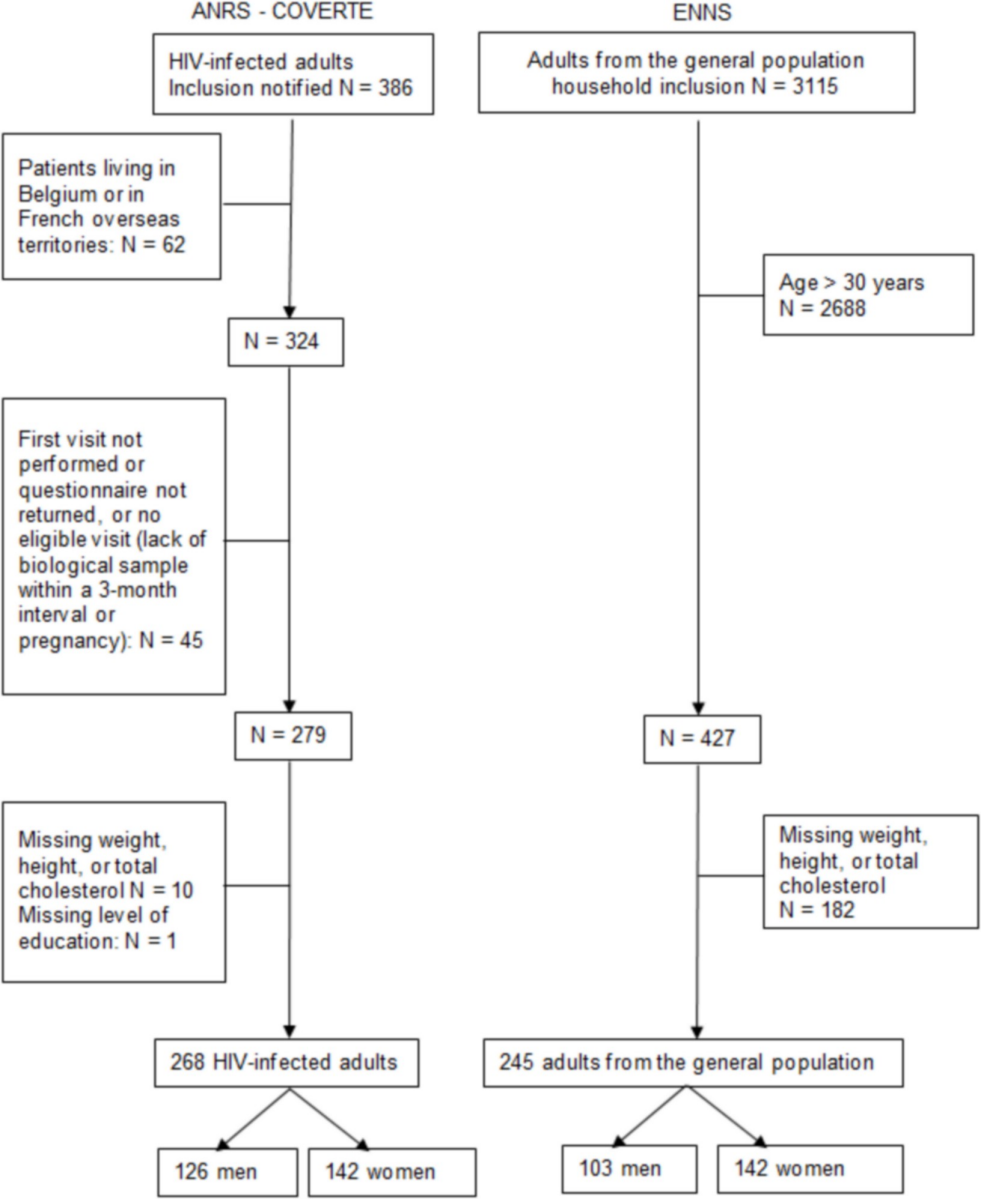
Diagram for sample selection from the ANRS-COVERTE CO19 cohort and ENNS study.

Between 2010 and 2016, the COVERTE cohort enrolled 386 patients infected with HIV since childhood, of which 268 were included in the analyses (Fig 1), consisting of 47% men, with a median age of 23 years [IQI: 21–26], of whom 55% completed high school. Their median BMI was 22.3 [IQI: 20.1–24.8]. The data used for these analyses were collected between 2013 and 2015 for 227 patients (85%). Among the 427 adults aged 18–30 years enrolled in the ENNS in 2006–2007, 245 were included in the analyses, consisting of 42% men, with a median age of 22.5 years [IQI: 19–26], of whom 53.9% completed high school. Their median BMI was 22.8 [IQI: 20.2–25.3].

Several differences between HIV-infected patients and the ENNS general population of the same gender remained after standardization based on education level and BMI (Table 1): HIV-infected patients were less likely to be born in France, to live in couple and to be employed or students, but tobacco use was similar; HIV-infected men were younger; and HIV-infected women were less likely to take hormonal contraception.

**Table 1:** Sociodemographic and behavioral characteristics of patients infected with HIV since childhood (ANRS COVERTE Cohort) and the French general population (ENNS).

	HIV-infected men (COVERTE: (N = 126)		Men from general population (ENNS: N = 103)	HIV-infected women (COVERTE: N = 142)		Women from general population (ENNS: N = 142)
	%	Stand. %*	%**	%	Stand. %*	%**
Age 25–30 (ref 18–24)	32.5	32.0	42.6	32.4	33.3	35.5
Education level <high school diploma	50.8	54.7	54.7	39.4	36.7	36.7
Employment	(10 missing data)			(15 missing data)		
- Not working	30.2	33.9	8.1	37.8	36.5	18.2
- Student	25.8	23.7	30.9	31.5	33.2	41.2
- Working	44.0	42.4	61.0	30.7	30.3	40.6
Born in metropolitan France	68.2	67.8	94.7	70.4	70.1	94.8
Married/living with a partner (ref: Single Separated/divorced/widowed)	(10 missing data)			(14 missing data)		
	17.2	16.9	32.1	24.2	23.5	41.4
Tobacco consumption	(1 missing data)			(1 missing data)		
- Every day	36.0	39.0	43.8	31.2	29.4	31.5
- Not every day	9.6	8.8	2.5	8.5	8.9	2.3
- In the past	12.8	12.9	12.0	14.2	14.9	14.2
- Never	41.6	39.3	41.8	46.1	46.8	52.0
Psychotropic drug use	3.2	3.5	3.3	4.9	4.8	3.2
Hormonal contraception	NA	NA	NA	21.8	21.6	49.3
Body mass index (BMI)						
- BMI < 18.5	7.9	6.9	0.5	9.8	9.6	10.2
- 18.5 ≤ BMI < 25	69.8	59.6	66.0	63.4	64.2	63.5
- BMI ≥ 25	22.2	33.5	33.5	26.8	26.2	26.2

NA: not applicable; Stand.: standardized

* standardized prevalence for education level and BMI ≥ 25

** prevalence estimates for general population from ENNS, taking into account the sampling design (unequal inclusion probabilities) and post-stratification to adjust each gender for the distribution of national census data on age, education, presence of at least one child in the household, season of medical examination, and the availability of weight, height, and total cholesterol measures.

At the time of the study visit, less than 1.5% of HIV-infected patients had never been treated while, in the others, the median duration of ART was 19 years (Table 2). Approximately 90% were currently on ART, two nucleoside reverse transcriptase inhibitors plus one protease inhibitor being the most frequent regimen (40%). Twelve patients were still on zidovudine, 12 others on didanosine and one on stavudine at the time of data collection. The median CD4 cell count was 554/microliter. Viral load was undetectable in 68% of patients, with a median of 3.19 log_10_ copies/mL, when detectable.

**Table 2:** Comparison of antiretroviral treatment and immune-virological status, according to gender, of patients infected with HIV since childhood from the ANRS CO19—COVERTE cohort.

	Men (N = 126)			Women (N = 142)			p-value
	N	%	M	N	%	M	
ART			0			0	0.181
- Never treated	2	1.6		1	0.7		
- Treatment stopped	5	4.0		13	9.2		
- On treatment	119	94.4		128	90.1		
Type of ART regimen			0			1	0.556
- 2NRTI + 1NNRTI	25	20.1		37	26.4		
- 2NRTI + PI	50	40.3		56	40.0		
- II + others	32	25.8		28	20.0		
- Other regimens	17	13.7		19	13.6		
CDC Stage C	33	26.2	0	29	20.4	0	0.264
Undetectable plasma viral load	81	68.6	8	93	67.4	4	0.830
	**Median**	**IQR**	**Miss**	**Median**	**IQR**	**Miss**	**p-value**
Viral load (log_10_ copies/mL), when detectable	3.64	2.11–4.25	8	3.03	2.01–4.39	4	0.589
CD4 (cells/microliter)	559	372–755	3	552	399–704	4	0.926
Time on ART (years)	18.8	12.4–22.1	0	18.9	12.1–21.8	1	0.806
Duration of exposure to ART with known metabolic toxicity* (years)	13.7	8.7–17.6	0	12.5	6.2–17.5	1	0.315

M: missing data; IQR: interquartile range; ART: antiretroviral therapy; NRTI: nucleoside reverse transcriptase inhibitors; NNRTI: non-nucleoside reverse transcriptase inhibitors; PI: protease inhibitors; II: Integrase Inhibitors

* stavudine, didanosine, zidovudine, protease inhibitors

Six HIV-infected participants were treated with blood pressure-lowering drugs, two with lipid-lowering drugs, and one with insulin for type 1 diabetes, whereas no participant in the ENNS survey received any of these drugs.

### Comparison of continuous metabolic parameters between HIV-infected patients and the general population

After adjustment for education level and being overweight, HIV-infected patients of both genders differed from the general population for several metabolic parameters (Table 3). They had higher waist-to-hip ratios than their counterparts from the general population, with higher mean waist circumference in HIV-infected women. Regarding the lipid profile, HIV-infected men showed a higher mean level of triglycerides and a lower mean level of HDL-cholesterol than those from men from the general population. Although their LDL-cholesterol mean levels were also lower, their total-to-HDL-cholesterol ratio tended to be higher than that from ENNS men. HIV-infected women had higher mean systolic blood pressure than ENNS women, and their mean total and HDL- and LDL- fractions of cholesterol were lower, with non significantly different mean cholesterol total-to-HDL ratio and triglycerides levels. However, the mean triglycerides-to-HDL cholesterol ratio was significantly higher in HIV-infected women. In addition, after further adjustment for the use of hormonal contraception, mean total and LDL-cholesterol were no longer different between HIV-infected and ENNS women (p = 0.176 and 0.120 respectively), whereas triglycerides levels were significantly higher (p = 0.021) and HDL-cholesterol still tended to be lower (p = 0.069) in HIV-infected women.

**Table 3:** Comparison of metabolic parameters of patients infected with HIV since childhood (COVERTE) versus the general population (ENNS), adjusted for being overweight and education level, by linear regression.

	HIV-infected men (COVERTE: N = 126)			Men from general population (ENNS: N = 103)			p-value^b^	HIV-infected women (COVERTE: N = 142)			Women from general population (ENNS: N = 142)			p-value^c^	p-value^d^
	Mean	95%CI	M	Mean^a^	95%CI	M		Mean	95%CI	M	Mean^a^	95%CI	M		
BMI	22.6	21.9–23.2	0	24.1	22.8–25.4	0	**0.026**	22.9	22.2–23.7	0	23.5	22.2–24.9	0	0.409	0.241
Waist/Hip ratio	0.91	0.88–0.93	22	0.86	0.85–0.89	1	**<10**^**−4**^	0.86	0.83–0.89	18	0.76	0.75–0.78	1	**<10**^**−4**^	**<10**^**−4**^
Waist circumference (cm)	81.0	79.1–82.9	22	84	80–88	1	0.419	80	77–83	17	76	73–78	1	**0.002**	**0.016**
Systolic blood pressure (mmHg)	122	120–125	10	120	117–124	0	0.088	116	113–119	10	109	107–112	0	**<10**^**−3**^	**<10**^**−4**^
Diastolic blood pressure (mmHg)	72	70–74	10	71	69–74	0	0.593	71	69–74	10	72	69–75	0	0.844	0.919
Fasting glucose (mmol/L)	4.7	4.6–4.9	3	4.9	4.7–5.1	1	0.117	4.6	4.5–4.8	1	4.6	4.5–4.8	7	0.914	0.765
Triglycerides (mmol/L)	1.4	1.1–1.7	1	1.1	0.9–1.2	0	**0.003**	1.0	0.9–1.2	1	0.9	0.8–1.1	0	0.311	**0.021**
Total cholesterol, (mmol/L)	4.2	4.0–4.5	0	4.4	4.2–4.7	0	0.226	4.5	4.2–4.7	0	4.8	4.5–5.0	0	**0.030**	**0.176**
LDL-cholesterol, (mmol/L)	2.4	2.2–2.6	5	2.6	2.5–2.8	7	**0.044**	2.5	2.3–2.7	6	2.8	2.6–3.0	4	**0.045**	**0.120**
HDL-cholesterol, (mmol/L)	1.2	1.1–1.3	4	1.3	1.2–1.5	2	**0.033**	1.4	1.3–1.5	5	1.5	1.4–1.7	2	**0.014**	**0.069**
Non-HDL cholesterol, (mmol/L)	3.0	2.8–3.3	4	3.1	2.9–3.3	2	0.673	3.0	2.8–3.2	5	3.2	3.0–3.5	2	0.093	0.326
Total to HDL-cholesterol ratio	3.8	3.5–4.1	4	3.5	3.3–3.8	2	0.059	3.3	3.1–3.5	5	3.2	3.0–3.5	2	0.343	0.158
Triglycerides to HDL-cholesterol ratio	1.4	1.1–1.8	5	0.9	0.7–1.1	2	**0.003**	0.8	0.7–0.9	6	0.6	0.6–0.7	2	**0.008**	**0.0008**

CI: Confidence Interval; M: missing data; BMI: Body Mass Index; HDL: high-density lipoprotein; LDL: low-density lipoprotein

^**a**^ weighted to take into account unequal inclusion probabilities and post-stratification according to each gender for national census data on age, education, presence of at least one child in the household, season of medical examination, and the availability of weight, height, and total cholesterol measures.

^**b**^ Comparisons between HIV-infected men and men from the general population using linear regression adjusted for education level and being overweight (except for BMI adjusted on education level only), taking into account sampling weights.

^**c**^ Comparisons between HIV-infected women and women from the general population using linear regression adjusted for education level and being overweight (except for BMI adjusted on education level only), taking into account sampling weights.

^**d**^ Comparisons between HIV-infected women and women from the general population using linear regression adjusted for education level, being overweight and use of oral contraceptives (except for BMI adjusted on education level only), taking into account sampling weights.

### Comparison of metabolic abnormalities between HIV-infected patients and the general population

An increased prevalence of several metabolic abnormalities was observed in COVERTE as compared to the ENNS population, after standardization or adjustment for education level and overweight.

HIV-infected men had a higher prevalence of elevated triglycerides and reduced HDL-cholesterol levels than men of the general population (Table 4). The prevalence of metabolic syndrome did not differ significantly: 13.2% (95%CI 7.1–19.2) versus 10.6% (95%CI 1.5–19.7). The most common cluster of abnormalities in participants with metabolic syndrome was elevated triglycerides, reduced HDL-cholesterol and elevated blood pressure, found in six out of 14 HIV-infected individuals (43%) and in 36% of those from the general population.

**Table 4:** Comparison of standardized prevalence of metabolic abnormalities in patients infected with HIV since childhood versus the general population for men (COVERTE and ENNS studies) adjusted for being overweight and education level, by logistic regression.

	HIV-infected men (COVERTE: n = 126)					General population (ENNS: n = 103)			Full logistic regression ^c^		
	N	%	Stand.%^a^	95%CI	M	%^b^	95%CI	M	aOR	95%CI	p-value
Overweight	28	22.2	22.2	14.9–29.5	0	33.5	21.2–45.8	0	0.6	0.3–1.1	0.106
Elevated blood pressure	41	35.0	36.1	27.2–45.0	9	31.0	19.2–42.8	0	1.6	0.8–3.3	0.193
Elevated waist circumference	12	11.4	16.2	9.0–23.4	21	14.3	5.2–23.3	1	1.5	0.4–5.2	0.497
Elevated fasting glucose	10	8.1	9.8	4.1–15.4	3	14.9	4.9–24.8	1	0.5	0.2–1.5	0.245
**Elevated Triglycerides**	**28**	**22.4**	**22.9**	**15.3–30.5**	**1**	**9.5**	**1.0–18.0**	**0**	**4.4**	**1.7–11.7**	**0.003**
**Reduced HDL-cholesterol**	**43**	**34.9**	**36.3**	**27.4–45.2**	**3**	**23.7**	**11.0–36.5**	**2**	**2.1**	**0.9–5.0**	**0.083**
Elevated LDL-cholesterol	13	10.7	11.5	5.5–17.4	4	12.4	6.0–18.7	7	0.9	0.4–2.0	0.735
Elevated total cholesterol	21	16.7	17.8	10.8–24.9	0	14.6	7.6–21.7	0	1.2	0.6–2.5	0.646
Metabolic syndrome	14	11.1	13.2	7.1–19.2	0	10.6	1.5–19.7	0	2.1	0.6–7.9	0.252

N: number; M: missing data; Stand.: standardized; aOR: adjusted Odds Ratio; CI: confidence interval; HDL: high-density lipoprotein; LDL: low-density lipoprotein

^**a**^ standardized on education level and body mass index ≥ 25 (except for overweight standardized on education level only)

^**b**^ weighted to take into account unequal inclusion probabilities and post-stratification, according to each gender for national census data on age, education, presence of at least one child in the household, season of medical examination, and the availability of weight, height, and total cholesterol measures

^**c**^ using logistic regression adjusted for education level and being overweight (except for overweight adjusted on education level only), taking into account sampling weights

HIV-infected women were more likely to have reduced HDL-cholesterol levels and a high waist circumference, although they were less likely to have elevated total cholesterol than women from the general population (Table 5). Their prevalence of metabolic syndrome was significantly higher than in women from the general population: 10.4% (95%CI 5.4–15.3) versus 1.7% (95%CI 0–4.1), respectively, with an adjusted odds ratio of 9.9 (95%CI 1.9–51.3, p = 0.006). After further adjustment for the use of oral contraceptives, odd-ratios related to cholesterol levels were no longer significant although other results were not modified. The most common cluster of abnormalities in the 15 HIV-infected women with metabolic syndrome was the combination of elevated triglycerides, reduced HDL-cholesterol and high waist circumference (n = 8). The three women with metabolic syndrome from the general population presented with different clusters but all had a high waist circumference.

**Table 5:** Comparison of standardized prevalence of metabolic abnormalities in patients infected with HIV since childhood versus the general population for women (COVERTE and ENNS), adjusted for being overweight and education level, by logistic regression.

	HIV-infected women (COVERTE: n = 142)					General population (ENNS: n = 142)			Logistic regression #1^c^			Logistic regression #2^d^		
	N	%	Stand. %^a^	95%CI	M	%^b^	95%CI	M	aOR	95%CI	p-value	aOR	95%CI	p-value
Overweight	36	25.3	25.4	18.2–32.5	0	26.2	16.3–36.2	0	0.9	0.4–1.8	0.831	0.8	0.3–1.7	0.601
Elevated blood pressure	29	22.0	21.1	14.2–28.1	10	13.6	5.8–21.5	0	1.8	0.8–4.1	0.129	1.9	0.8–4.8	0.153
**Elevated waist circumference**	**51**	**40.8**	**40.3**	**33.5–47.2**	**17**	**23.9**	**14.3–33.6**	**2**	**5.9**	**2.2–15.6**	**0.0004**	**4.1**	**1.5–11.3**	**0.007**
Elevated fasting glucose	7	5.0	5.7	1.8–9.6	1	1.2	0–3.6	7	ND	ND	ND	ND	ND	ND
Elevated Triglycerides	13	9.2	8.8	4.3–13.2	1	7.9	1.2–14.7	0	1.1	0.3–3.5	0.862	2.0	0.6–6.1	0.198
**Reduced HDL-cholesterol**	**55**	**40.1**	**39.0**	**31.0–47.0**	**5**	**24.9**	**15.0–34.8**	**2**	**2.0**	**1.0–4.0**	**0.034**	**1.8**	**0.8–3.8**	**0.138**
Elevated LDL-cholesterol	19	14.0	14.1	8.1–20.1	6	16.8	8.7–25.0	4	0.8	0.3–1.7	0.557	1.0	0.4–2.2	0.988
**Elevated total cholesterol**	**28**	**19.7**	**20.6**	**13.7–27.5**	**0**	**32.4**	**22.4–42.4**	**0**	**0.5**	**0.3–1.0**	**0.036**	**0.6**	**0.3–1.3**	**0.192**
**Metabolic syndrome**	**15**	**10.6**	**10.4**	**5.4–15.3**	**0**	**1.7**	**0–4.1**	**0**	**9.9**	**1.9–51.3**	**0.006**	**9.4**	**1.7–52.1**	**0.010**

N: number; M: missing data; Stand.: standardized; aOR: adjusted Odds Ratio; CI: confidence interval; HDL: high-density lipoprotein; LDL: low-density lipoprotein

^**a**^ standardized on education level and body mass index ≥ 25 (except for overweight standardized on education level only)

^**b**^ weighted to take into account unequal inclusion probabilities and post-stratification, according to each gender for national census data on age, education, presence of at least one child in the household, season of medical examination, and the availability of weight, height, and total cholesterol measures

^**c**^ using logistic regression adjusted for education level and being overweight (except for overweight adjusted on education level only), taking into account sampling weights

^**d**^ using logistic regression adjusted for education level, being overweight and use of oral contraceptives (except for overweight adjusted on education level only), taking into account sampling weights

ND: not done because of too few events, the model was overfitted

### Sensitivity analyses

We conducted sensitivity analyses on the 412 subjects born in France (186 HIV-infected patients, including 86 men, and 232 adults from the general population, including 94 men). The results were quite similar to those obtained with the whole sample, except for a stronger association with reduced HDL-cholesterol in HIV-infected men and a weaker association with elevated total cholesterol in HIV-infected women (S1 Table).

## Discussion

This study is the first to compare the metabolic parameters of young adults who acquired HIV infection during childhood and controls of the same age from the general population. It points to increased prevalence of cardiovascular and insulin resistance risk factors in HIV-infected young subjects.

HIV-infected men had a lower BMI, but higher waist-to-hip ratio, higher triglycerides and lower HDL-cholesterol levels (without showing increased LDL-cholesterol and non-HDL cholesterol levels), than men of the same age in the general population. HIV-infected women had a similar BMI as women of the same age from the general population, but a higher waist-to-hip ratio associated with a larger waist circumference, and a higher systolic blood pressure. They also had lower HDL-cholesterol levels without increased LDL-cholesterol and non-HDL cholesterol levels. They had a higher mean triglycerides-to-HDL-cholesterol ratio and a higher prevalence of the metabolic syndrome than women in the general population. In addition, after further adjustment on the use of hormonal contraceptives, HIV-infected women showed significantly higher triglycerides mean levels than those from the general population. These results indicate that 18 to 30 year-olds with HIV infection display an atherogenic metabolic profile, as compared to uninfected adults of the same age, similar to the findings in French HIV-infected patients aged 35–44 years on protease inhibitors (APROCO study) [10]. However, in the latter study [10], the prevalence of smoking was much higher than in the general population, partially explaining the higher cardiovascular risk. This was not the case in our study, since tobacco consumption was similar between the two young populations.

Higher waist-to-hip ratio and serum triglycerides, associated with lower HDL-cholesterol levels and higher triglycerides-to-HDL-cholesterol than in the general population, without difference in total or LDL cholesterol levels, are highly suggestive of a state of lower insulin sensitivity in young subjects with HIV infection since childhood [25]. This metabolic profile was also observed in HIV-infected adults in the APROCO study, exposed to first generation antiretrovirals. Consistently, the young adult patients in our study have also been exposed for several years to first generation ART regimens, including stavudine, zidovudine, didanosine, and protease inhibitors, such as ritonavir, known to alter lipid and glucose metabolism via adipocyte dysfunction, inducing lipodystrophy insulin resistance, and dyslipidemia [26].

In the aforementioned study of HIV-infected French adults aged 35–44 years [10], metabolic profiles were similar between men and women. In our study of young adults exposed to HIV infection and ART since childhood, we observed distinct gender-specific metabolic features with higher triglyceride levels in HIV-infected men, and larger waist circumference in HIV-infected women, as compared to the general population of same age. This was not explained by differences in antiretroviral exposure, clinical, or immuno-virological status at the time of the study, but more likely by sex and gender-related factors. First, sex-based differences in immune activation have been described with a stronger interferon alpha response in women [27] possibly contributing to metabolic disorders. Second, HIV infection and antiretroviral treatments may interact with the estrogen/testosterone balance promoting androgen exposure, resulting in an increase of visceral abdominal fat and lipid concentrations [21, 28, 29], with more visible effects in women. Finally, differences in dietary and lifestyle habits such as alcohol consumption, physical activity and psychological status [21], might also explain an effect of gender on the associations between HIV and metabolic abnormalities. Also, the risk of polycystic ovary syndrome, strongly associated with truncal adiposity in the general population, was not reported to be increased in HIV-infected women with a mean age of 41 years [30]. Further study of the ovarian and reproductive consequences of long-term exposure to insulin resistance risk factors, as observed in young women with HIV infection since childhood, would be of interest. Importantly, in addition to a proper screening of cardiometabolic diseases over time, the knowledge of increased prevalence of insulin resistance risk factors in HIV-infected young women reinforces the need for a proper evaluation of their lipid profile before prescribing a contraceptive pill.

Our study has several strengths. First, the availability of two national French studies that collected metabolic and behavioral data for a similar number of eligible subjects at comparable time periods made it possible to compare young HIV-infected patients with a general population of the same gender and age distribution. Second, it was possible to standardize prevalence and adjust models for BMI, strongly associated with metabolic syndrome, and education level, associated with behavioral factors, nutritional habits, and metabolic syndrome in women in a previous ENNS analysis [31]. We did not standardize for the distribution of age, as the 18 to 30-year age group appeared to be very homogenous, with a low global risk for metabolic syndrome.

Our study has several limitations. First, the general population, as non-HIV-exposed group was assumed rather than systematic HIV testing, which might have underestimated the observed associations. The prevalence of HIV in France being about 4/1000 habitants, this bias may be low [32]. Second, the current HIV-exposed group may not be representative of the whole population of young adults infected with HIV since childhood in France which may have introduced a selection bias. In particular, only one third of the patients from the Enquête Périnatale Française, ANRS CO1 were still followed-up in COVERTE. We can hypothesize that those who agreed to participate in COVERTE are those who were more concerned and adherent with medical follow-up and presented a better health status, which might have also underestimated the observed associations. Third, ethnic differences in the prevalence of metabolic syndrome have been reported in the general population, particularly in the United States [33], but ethnicity data were unavailable for our cohorts, and stratification by country of birth, as a proxy for ethnicity, was not possible because of the small number of ENNS participants born outside of France. We thus conducted a sensitivity analysis on men and women born in metropolitan France, which confirmed our study results. Fourth, we could not study direct indexes of glucose tolerance and insulin sensitivity, because oral glucose tolerance tests and insulin measurements were not performed in the ENNS study. Fifth, we could not compare behaviors concerning diet or physical activity as they were collected very differently in the two studied populations. Another concern is the risk of false positive associations due to multiple testing. We chose not to perform adjustments for multiple testing because such an approach is questionable from a methodological standpoint [34], particularly in such exploratory analysis.

Our results show that, as compared to the general population of the same age living in France, young adults infected with HIV since childhood did not display increased tobacco consumption, which represent the main modifiable behavioral cardiovascular risk factor. We can also note that fasting glucose, and LDL-cholesterol did not differ between the two populations. The current treatment, care and support for vertically HIV-infected patients may have contributed to maintain these favourable metabolic parameters. However, young HIV-infected adults had a higher prevalence of dyslipidemia and android fat distribution, which are strongly associated with an increased risk of insulin resistance and further cardiometabolic disease, even in the youngest HIV-infected patients [35]. This supports a close monitoring and longitudinal screening for the risk of cardiovascular diseases and diabetes in these patients. Similar attention should be drawn to cognitive dysfunction which has been associated with central obesity and diabetes in HIV patients [36, 37]. An ongoing long-term study will determine whether these increased risk factors correlate with alterations of functional cardiovascular markers in this cohort.

## Supporting information

S1 File The ANRS COVERTE-CO19 Study. (DOCX)

S2 File Selection of the COVERTE questionnaires, in French and in English. (DOC)

S3 File The ENNS Study. (DOCX)

S4 File Selection of the ENNS questionnaires, in French and in English. (DOC)

S1 Dataset Database for the present analysis. (XLSX)

S1 Table Sensitivity analysis: Comparison of prevalence of metabolic abnormalities of patients infected with HIV since childhood with the general population born in France (COVERTE and ENNS), adjusted for being overweight and education level, by logistic regression. (DOCX)
