# Supplementary material for: Metabolic risk factors in young adults infected with HIV since childhood compared with the general population
Source: PLoS One. 2018 Nov 8;13(11):e0206745. doi: 10.1371/journal.pone.0206745 (PMC6226109; doi:10.1371/journal.pone.0206745)
Supplement: S1 File — (DOCX) [file pone.0206745.s001.docx]

**S1 File. The ANRS COVERTE-CO19 Study**

**Project team**

Principal Investigators : Josiane Warszawski, Jean-Paul Viard.

Project managers : Nelly Briand, Mélanie Thoumine, Laura Nailler

Research monitors : Florie Chretien, Marine Pytkowski, Nadine Lubango, Feriel Ayat, Kamilia Asnoun

**Scientific Committee** : Appay V (Inserm U945, Paris), Blanche S (Hôpital Necker, Paris), Boccara F (Hôpital Saint Antoine, Paris), Boufassa F (Inserm CESP U1018, Le Kremlin-Bicêtre), Dollfus C (Hôpital Trousseau, Paris), Dray-Spira R (Inserm CESP U1018, Villejuif), Faye A (Hôpital Robert Debré, Paris), Funck-Brentano I (Hôpital Necker, Paris), Jeantils V (Hôpital Jean-Verdier, Bondy), Le Chenadec J (Inserm CESP U1018, Le Kremlin-Bicêtre), Levine M (Hôpital Robert-Debré, Paris), Meyer L (Hôpital Bicêtre, Le Kremlin-Bicêtre), Rouzioux C (Hôpital Necker, Paris), Trocme N (Hôpital Trousseau, Paris), Velter A (Institut National de Veille Sanitaire), Viard JP (Hôtel Dieu, Paris), Vigouroux C (Hôpital Saint-Antoine, Paris), Warszawski J (Hôpital Bicêtre, Le Kremlin-Bicêtre)

**Clinical centers (coordinator):** Perpignan (Aumaitre H), Lagny (Froguel E), Argenteuil (Genet P), Villeneuve Saint Georges (Patey O, Chace A), St Louis (Gerard L, Pintado C), St Etienne (Lucht F), Besançon (Chirouze C), Bordeaux -Hôpital Pellegrin (Dupon M, Neau D), Haut Lévêque (Pellegrin JL), Saint André (Morlat P, Mercie P)-, Amiens (Schmit JL), Bicêtre (Goujard C), Aix en Provence (Allegre T), Toulon (Lafeuillade A), Garches (De Truchis P), Jean Verdier (Jeantils V), Institut Pasteur (Duvivier C), Tenon (Canestri A), Avicenne (Bouchaud O), HEGP (Karmochkine M), Cochin (Salmon-Ceron D, Firtion G), Suresnes (Zucman D), Louis Mourier (Mortier E, Floch-Tudal C), Pitié Salpêtrière (Tubiana R, Simon A), St Antoine (Girard PM), Fort de France (Cabie A), Angers (Chennebault JM), Limoges (Weinbreck P), Marseille (Poizot-Martin I, Moreau J), Bichat Claude Bernard (Matheron S), Nantes (Reliquet V), La Roche/Yon (Perré P), Rennes (Arvieux C), Strasbourg (Cheneau C, Entz-Werle N), Tours (Bernard L), Toulouse (Delobel P, Tricoire J), Caen (Verdon R), Clermont Ferrand (Jacomet C, Dore E), Dijon (Piroth L), Tourcoing (Ajana F), Rouen (Debab Y), Compiègne (Merrien D), Lyon (Cotte L, Kebaili K, Makhloufi D), La Réunion (Poubeau P, Gaud C), Créteil (Garrait V), St Denis (Khuong MC, Bolot P), Mulhouse (Beck-Wirth G), Necker (Blanche S, Treluyer JM), Orléans (Prazuck T), Nîmes (Barbuat C), Hôtel Dieu-Paris (Viard JP, Girard T), Guadeloupe (Fernandes E), Chalon sur Saône (Martha B, Martha SA), Cayenne (Elenga N), Robert Debré (Faye A, Levine M), Armand Trousseau (Dollfus C), Lille (Mazingue F), St Germain en Laye (Cazenave B), Nancy (May T), Cergy-Pointoise (Blum L), Lorient (Niault M), Le Mans (Hitoto H), St Antoine (Boccara F)
