# Supplementary material for: Metabolic risk factors in young adults infected with HIV since childhood compared with the general population
Source: PLoS One. 2018 Nov 8;13(11):e0206745. doi: 10.1371/journal.pone.0206745 (PMC6226109; doi:10.1371/journal.pone.0206745)
Supplement: S2 File — (DOC) [file pone.0206745.s002.doc]

Version 1.4 – Mars 2011

**S2 File. Selection of the COVERTE questionnaires, in French and in English**

**Cohorte de jeunes adultes infectés par le VIH**

**par voie verticale ou pendant l’enfance**

**ANRS CO 19**

**COVERTE**

**Sélection du questionnaire « RECAPITULATIF DU SUIVI DU PATIENT**

**DEPUIS LE DIAGNOSTIC DE VIH »**

Date de naissance ⊥  ⊥   ⊥⊥

**Date du diagnostic d’infection à VIH A-t-il été fait en France ?** _0_ Non _1_ Oui

 ⊥  ⊥⊥    

**M**

**M**

**A**

**A**

**A**

**A**

**Date de 1^ère^ prise en charge en France**

 ⊥  ⊥⊥    

**M**

**M**

**A**

**A**

**A**

**A**

| **Antécédents d’événements classant stade CDC C** **_0_ Non** **_1_ Oui** | |
| --- | --- |
| **Préciser** | **Date de survenue** |
|  | ⊥  ⊥  ⊥⊥      **J**  **J**  **M**  **M**  **A**  **A**  **A**  **A** |
|  | ⊥  ⊥  ⊥⊥      **J**  **J**  **M**  **M**  **A**  **A**  **A**  **A** |
|  | ⊥  ⊥  ⊥⊥      **J**  **J**  **M**  **M**  **A**  **A**  **A**  **A** |
|  | ⊥  ⊥  ⊥⊥      **J**  **J**  **M**  **M**  **A**  **A**  **A**  **A** |
|  | ⊥  ⊥  ⊥⊥      **J**  **J**  **M**  **M**  **A**  **A**  **A**  **A** |
|  | ⊥  ⊥  ⊥⊥      **J**  **J**  **M**  **M**  **A**  **A**  **A**  **A** |
|  | ⊥  ⊥  ⊥⊥      **J**  **J**  **M**  **M**  **A**  **A**  **A**  **A** |
|  | ⊥  ⊥  ⊥⊥      **J**  **J**  **M**  **M**  **A**  **A**  **A**  **A** |

***(Classifications jointes)***

**RECAPITULATIF DES TRAITEMENTS ANTIRETROVIRAUX**

***Séquence thérapeutique** = traitement ou combinaison de traitements donnés simultanément pendant une période continue

Toute interruption thérapeutique de plus de 15 jours doit être notée comme une nouvelle séquence thérapeutique

| **Traitements donnés simultanément** | **Début** | **Fin** | **Motif d'arrêt ou de changement** |
| --- | --- | --- | --- |
| **1^ère^ séquence***  **-**  **-**  **-**  **-** | Date de début  ⊥  ⊥  ⊥⊥      **J**  **J**  **M**  **M**  **A**  **A**  **A**  **A** | 🞏_1_ En cours  ou  ⮩ Date de fin  ⊥  ⊥  ⊥⊥      **J**  **J**  **M**  **M**  **A**  **A**  **A**  **A** | 🞏_1_ Entrée dans un protocole  🞏_2_ Fenêtre thérapeutique  🞏_3_ Problème d’observance  🞏_4_ Problème d’efficacité🡪 Préciser    🞏_5_ Problème de toxicité🡪 Préciser    🞏_6_ Autre(s), Préciser |
| **2^ème^ séquence***  **-**  **-**  **-**  **-** | Date de début  ⊥  ⊥  ⊥⊥      **J**  **J**  **M**  **M**  **A**  **A**  **A**  **A** | 🞏_1_ En cours  ou  ⮩ Date de fin  ⊥  ⊥  ⊥⊥      **J**  **J**  **M**  **M**  **A**  **A**  **A**  **A** | 🞏_1_ Entrée dans un protocole  🞏_2_ Fenêtre thérapeutique  🞏_3_ Problème d’observance  🞏_4_ Problème d’efficacité🡪 Préciser    🞏_5_ Problème de toxicité🡪 Préciser    🞏_6_ Autre(s), Préciser |
| **3^ème^ séquence***  **-**  **-**  **-**  **-** | Date de début  ⊥  ⊥  ⊥⊥      **J**  **J**  **M**  **M**  **A**  **A**  **A**  **A** | 🞏_1_ En cours  ou  ⮩ Date de fin  ⊥  ⊥  ⊥⊥      **J**  **J**  **M**  **M**  **A**  **A**  **A**  **A** | 🞏_1_ Entrée dans un protocole  🞏_2_ Fenêtre thérapeutique  🞏_3_ Problème d’observance  🞏_4_ Problème d’efficacité🡪 Préciser    🞏_5_ Problème de toxicité🡪 Préciser    🞏_6_ Autre(s), Préciser |
| **4^ème^ séquence***  **-**  **-**  **-**  **-** | Date de début  ⊥  ⊥  ⊥⊥      **J**  **J**  **M**  **M**  **A**  **A**  **A**  **A** | 🞏_1_ En cours  ou  ⮩ Date de fin  ⊥  ⊥  ⊥⊥      **J**  **J**  **M**  **M**  **A**  **A**  **A**  **A** | 🞏_1_ Entrée dans un protocole  🞏_2_ Fenêtre thérapeutique  🞏_3_ Problème d’observance  🞏_4_ Problème d’efficacité🡪 Préciser    🞏_5_ Problème de toxicité🡪 Préciser    🞏_6_ Autre(s), Préciser |

**Sélection du questionnaire « INCLUSION»**

**CARACTERISTIQUES SOCIO-DEMOGRAPHIQUES**

**Sexe**

1 Masculin _2_ Féminin

**Lieu de naissance du patient**

Commune Département |__|__|__| 

Si France métropolitaine, préciser la maternité de naissance :

Pays de naissance (si hors France)

**TRAITEMENT ANTIRETROVIRAL ACTUEL**

**Le patient a-t-il été traité avant l’inclusion ?**

0 Non, jamais traité _1_ Oui

**Si oui**, dernière séquence thérapeutique prescrite = antirétroviraux prescrits ou renouvelés lors de la précédente consultation (noter les différents antirétroviraux de cette séquence) :

- -

- -

**Date de début (de la dernière séquence) :**

⊥  ⊥  ⊥⊥    

**J**

**J**

**M**

**M**

**A**

**A**

**A**

**A**

**Date de fin :**  ou  traitement toujours en cours

⊥  ⊥  ⊥⊥    

**J**

**J**

**M**

**M**

**A**

**A**

**A**

**A**

**Consultation d’inclusion**

Est-ce qu’un traitement est prévu à l’issue de la consultation d’inclusion ? _0_ Non_1_ Oui

**Si oui**, _1_ Poursuite du traitement prescrit lors de la consultation précédente

0 Nouveau traitement (modification partielle ou totale)

🡪 **Si un nouveau traitement est prescrit**: **noter les différents antirétroviraux prescrits à l’issue de cette consultation (donnés simultanément)**

- -

- -

- -

**Date de début :**

⊥  ⊥  ⊥⊥    

**J**

**J**

**M**

**M**

**A**

**A**

**A**

**A**

**Nombre total de prises :** |__|__| / 24 h

**Si aucun traitement n’est prescrit**, préciser :

0 Pas d’indication

1 Refus du patient

2 Autre raison, Préciser

**AUTRES TRAITEMENTS EN COURS AU MOMENT DE L’INCLUSION**

**Traitements hypolipémiants, hypoglycémiants, antihypertenseur (et autres cardiovasculaires)** **_0_ Non** **_1_ Oui**

**Si oui**, lequel :

Nom du traitement Date de début

⊥  ⊥⊥ 

**M**

**M**

**A**

**A**

**A**

**A**

⊥  ⊥⊥ 

**M**

**M**

**A**

**A**

**A**

**A**

**Traitements par psychotropes** **_0_ Non** **_1_ Oui**

**Si oui**, lequel :

Nom du traitement Date de début

⊥  ⊥⊥  

**M**

**M**

**A**

**A**

**A**

**A**

⊥  ⊥⊥ 

**M**

**M**

**A**

**A**

**A**

**A**

**Autres traitements** **_0_ Non** **_1_ Oui**

**Si oui**, lequel :

Nom du traitement Date de début

⊥  ⊥⊥ 

**M**

**M**

**A**

**A**

**A**

**A**

⊥  ⊥⊥ 

**M**

**M**

**A**

**A**

**A**

**A**

**Sélection du questionnaire médical (inclusion et visites suivantes)**

**Date de l’examen clinique:**

⊥  ⊥  ⊥⊥    

**J**

**J**

**M**

**M**

**A**

**A**

**A**

**A**

**Mesures anthropométriques**

**Poids** _|__|__ kg **Taille** |__|__|__ cm

**Périmètre taille**^(a)^ |__|__|__ cm **Périmètre Hanche**^(a)^ |__|__|__ cm

**Examen cardio-vasculaire**

- Pression artérielle ___|  / ____| mm Hg

| **Antécédents obstétricaux** |  |  |
| --- | --- | --- |
| **Grossesse en cours ?** _0_ Non _1_ Oui |  |  |

**Antécédents de pathologies des stades CDC C**

**Pathologie classante C** _0_ **Non** _1_ **Oui**

**Si oui**, date de passage en stade C :  

⊥  ⊥  ⊥⊥    

**J**

**J**

**M**

**M**

**A**

**A**

**A**

**A**

Première pathologie présente au moment du passage :

**Au moment de la consultation, le patient présente-t-il :**

**Une pathologie classante C** **_0_ Non** **_1_ Oui**

**En cours** **_0_ Non** **_1_ Oui**

**Si oui**, préciser : Date de survenue

⊥  ⊥  ⊥⊥    

**J**

**J**

**M**

**M**

**A**

**A**

**A**

**A**

préciser : Date de survenue

⊥  ⊥  ⊥⊥    

**J**

**J**

**M**

**M**

**A**

**A**

**A**

**A**

**Le patient est-il sous traitement antirétroviral ?** **_0_ Non** **_1_ Oui**

Si non, préciser

0 Pas d’indication

1 Refus du patient

2 Autre raison, Préciser :

| **Dernière séquence reportée dans le dernier questionnaire COVERTE reçu le** ⊥  ⊥  ⊥⊥  **J**  **J**  **M**  **M**  **A**  **A**  **A**  **A**  **Il s’agissait de** _0_ aucun traitement  **OU**   - - - -   Cette dernière séquence avait débuté le ⊥ ⊥ ⊥⊥  **J**  **J**  **M**  **M**  **A**  **A**  **A**  **A**  Y a t-il eu un changement depuis cette date ? _0_ Non_1_ Oui  **Si oui,** noter la date de fin de cette séquence ⊥ ⊥ ⊥⊥   **J**  **J**  **M**  **M**  **A**  **A**  **A**  **A**  Motif :  **Noter la ou les séquence(s) thérapeutique(s) ultérieure(s) dans le tableau ci-dessous, y compris celles prescrites lors de cette consultation :** | | | |
| --- | --- | --- | --- |
| **Traitements donnés simultanément** | **Début** | **Fin** | **Motif d'arrêt ou de changement** |
| **1^ère^ séquence***  **-**  **-**  **-**  **-** | Date de début  ⊥  ⊥  ⊥⊥      **J**  **J**  **M**  **M**  **A**  **A**  **A**  **A** | 🞏_1_ En cours  ou  ⮩ Date de fin  ⊥  ⊥  ⊥⊥      **J**  **J**  **M**  **M**  **A**  **A**  **A**  **A** | 🞏_1_ Entrée dans un protocole  🞏_2_ Fenêtre thérapeutique  🞏_3_ Problème d’observance  🞏_4_ Problème d’efficacité🡪 Préciser    🞏_5_ Problème de toxicité🡪 Préciser    🞏_6_ Autre(s), Préciser |
| **2^ème^ séquence***  **-**  **-**  **-**  **-** | Date de début  ⊥  ⊥  ⊥⊥      **J**  **J**  **M**  **M**  **A**  **A**  **A**  **A** | 🞏_1_ En cours  ou  ⮩ Date de fin  ⊥  ⊥  ⊥⊥      **J**  **J**  **M**  **M**  **A**  **A**  **A**  **A** | 🞏_1_ Entrée dans un protocole  🞏_2_ Fenêtre thérapeutique  🞏_3_ Problème d’observance  🞏_4_ Problème d’efficacité🡪 Préciser    🞏_5_ Problème de toxicité🡪 Préciser    🞏_6_ Autre(s), Préciser |
| **3^ème^ séquence***  **-**  **-**  **-**  **-** | Date de début  ⊥  ⊥  ⊥⊥      **J**  **J**  **M**  **M**  **A**  **A**  **A**  **A** | 🞏_1_ En cours  ou  ⮩ Date de fin  ⊥  ⊥  ⊥⊥      **J**  **J**  **M**  **M**  **A**  **A**  **A**  **A** | 🞏_1_ Entrée dans un protocole  🞏_2_ Fenêtre thérapeutique  🞏_3_ Problème d’observance  🞏_4_ Problème d’efficacité🡪 Préciser    🞏_5_ Problème de toxicité🡪 Préciser    🞏_6_ Autre(s), Préciser |

***Séquence thérapeutique** = traitement ou combinaison de traitements donnés simultanément pendant une période continue. Toute interruption thérapeutique de plus de 15 jours doit être notée comme une nouvelle séquence thérapeutique.

**Traitements hypolipémiants, hypoglycémiants, antihypertenseur (et autres cardiovasculaires)** **_0_ Non** **_1_ Oui**

**Si oui**, lequel

Nom du traitement Date de début si nouveau EN COURS Date d’arrêt éventuel

 ⊥ ⊥ ⊥⊥  **_1_**         ⊥ ⊥ ⊥⊥

 ⊥ ⊥ ⊥⊥  **_1_** ⊥ ⊥ ⊥⊥

**Traitements par psychotropes** **_0_ Non** **_1_ Oui**

**Si oui**, lequel

Nom du traitement Date de début si nouveau EN COURS Date d’arrêt éventuel

 ⊥ ⊥ ⊥⊥  **_1_** ⊥ ⊥ ⊥⊥

 ⊥ ⊥ ⊥⊥  **_1_** ⊥ ⊥ ⊥⊥

**Autres traitements** **_0_ Non** **_1_ Oui**

**Si oui**, lequel

Nom du traitement Date de début si nouveau EN COURS Date d’arrêt éventuel

 ⊥ ⊥ ⊥⊥  **_1_** ⊥ ⊥ ⊥⊥

 ⊥ ⊥ ⊥⊥  **_1_** ⊥ ⊥ ⊥⊥

**Sélection bilan biologique**

**Marqueurs lymphocytaires Date*:**

⊥  ⊥  ⊥⊥    

**J**

**J**

**M**

**M**

**A**

**A**

**A**

**A**

CD4  /mm^3^ et %

**Bilan métabolique Date[[1]](#footnote-2) :**

⊥  ⊥  ⊥⊥    

**J**

**J**

**M**

**M**

**A**

**A**

**A**

**A**

Glycémie  ,  mmol/l ou  ,  g/l

Cholestérol total  ,  mmol/l ou  ,  g/l

HDL cholestérol  ,  mmol/l ou  ,  g/l

LDL cholestérol  ,  mmol/l ou  ,  g/l **_1_ calculée** **_2_ mesurée**

(Ou <  ,  mmol/l ou <  ,  g/l)

Triglycérides  ,  mmol/l ou  ,  g/l

**Quantification ARN VIH-1**

**Date du prélèvement[[2]](#footnote-3)** :

⊥  ⊥  ⊥⊥    

**J**

**J**

**M**

**M**

**A**

**A**

**A**

**A**

**Technique** :

1 Roche Cobas Taqman 2

2 ABBOTT Real Time

3 Autres, précisez :

**Résultat**

0 < seuil **🡪**  Seuil (copies/ml) :  ou :  ,  log_10_

1 ≥ seuil

Nombre de copies d’ARN-VIH-1/ml :   

ou :  ,  log_10_

ou :  ,  log_10_

**Sélection de l’auto-questionnaire**

V1.4

**Quelle est actuellement votre situation de famille ?**

**2.**

Célibataire

Marié(e)

Pacsé(e)

Concubinage

Divorcé(e) ou en instance de divorce

Veuf (ve)

**Quel est votre pays de naissance ?**

**7.**

France métropolitaine

DOM/TOM, préciser :

Autre pays, préciser :

**9. Votre résidence principale est actuellement :**

En France

Dans un autre pays

**Quel est le diplôme le plus élevé que vous ayez obtenu ?**

**11.**

Vous n’avez pas de diplôme

**Dernière classe fréquentée** :

Vous avez un diplôme

**🡪 Lequel ?**

Brevet des collèges

Certificat d’Aptitude Professionnelle (CAP) ou équivalent

Brevet d’études professionnelles (BEP)

Baccalauréat technologique

Baccalauréat professionnel

Baccalauréat général

Niveau Bac + 2 (BTS, DUT, DEUG) ou équivalent

Niveau Bac + 3 (Licence) ou équivalent

Niveau Bac + 4 ou 5 (Master, Maîtrise, DEA, DESS, Ingénieur,…) ou équivalent

Doctorat ou équivalent

Autre diplôme, préciser :

**.**

**Actuellement, concernant votre activité professionnelle :**

Vous ne travaillez pas

**🡪 Vous êtes** :

Etudiant, lycéen

A la recherche de votre premier emploi

Au chômage

En congé maladie longue durée (depuis plus de 6 mois)

En allocation adulte handicapé ou pension d’invalidité

En congé parental

Père/Mère au foyer

Autre situation, préciser :

Vous travaillez (ou vous êtes en congé maladie court ou congé maternité)

**44.**

**Avez-vous déjà fumé une cigarette ?**

Non

Oui

- **Actuellement vous fumez :**

Tous les jours

Pas tous les jours

Vous ne fumez plus

1. ?Si la date est différente de la date du bilan biologique [↑](#footnote-ref-2)
2. Si la date est différente de la date du bilan biologique [↑](#footnote-ref-3)
