# Supplementary material for: Metabolic risk factors in young adults infected with HIV since childhood compared with the general population
Source: PLoS One. 2018 Nov 8;13(11):e0206745. doi: 10.1371/journal.pone.0206745 (PMC6226109; doi:10.1371/journal.pone.0206745)
Supplement: S3 File — (DOCX) [file pone.0206745.s003.docx]

**S3 File. The ENNS Study**

**Project team**

Principal Investigators : Katia Castetbon, Serge Hercberg.

Epidemiologists : Valérie Deschamps, Benoît Salanave, Michel Vernay.

Project manager : Aurélie Malon.

Research monitors : Sophie Carles, Amivi Oleko, Candice Roudier, Emmanuelle Szego.

Assistant : Isabelle Jaeglé.

**Dieticians :** G. Alavin, A. Aubert, C. Bertiner, I. Descamps, D. Doucet, B. Duplessis, D. Elie, E. Milcent, D. Albessard, G. Dubois, E. Gomes, F. Guenard, L. Guerin, H. Le Roux, M. Talon, E. Tisseron, C. Debliqui, AC. De Le Vallee, S. Denoyelle, S. Kamenney, A. Lebrasseur, C.Meignan, N. Pottier, MN. Bernard Leprince, M. Maugeais, M. Piron, L. Rouger, C. Andriot, A. Chennesseau, N. Dambreville, D. Baudet, D. Cassin, E. Lacroix, C. Leglise Blanchard, E. Bertrand, V. Delaroque, D. Rome, FI. Saccoccio Franzini, G. Veronese, A. Baur, K. Bernard, D. Maitrot, S. Poupeney, N. Gillot, C. Robert, S. Saas

**Physicians :** G. Debrus, H. Dehiri, D. Djoughi, M. Kahina Belkacem, V. Pechtner, P. Ralaimazava, C. Rault, W. Tabet Aoul.

**Nurses :** S. Artur, E. Bevilacqua, A. Chevallier, P. De Massey, F. Durand, P. Ferralis, A. Gitton, C. Hurbourg, C. Jacquot-Bastien, L. Keller, R. Lesage, E. Lodewyckx, P. Manquin, MH. Martinet Gourdon, AC. Merino, F. Michen, D. Mourichou, J. Pernot, C. Phillipe, G. Picot, C. Piednoir, C. Piergiorgi, N. Piverd, M. Poulette, C. Raoux, C. Veyssiere, V. Vivies Andrieu, P. Yagoubi, JM. Wallon

**Medical examination centres (CnamTS) :** CES de Paris CPAM, CES de Paris IPC, CES de Bobigny, CES de Melun, CES de Meaux, CES de Tourcoing, CES de Lille Institut Pasteur, CES de Roubaix, CES de Douai, CES de Valenciennes, CES de Cambrai, CES de Saint Quentin, CES d’Amiens, CES de Creil, CES du Havre, CES de Saint Lo, CES de Herouville Saint Clair (Caen), CES d’Alençon, CES de Dunkerque, CES de Crépy-en-Valois, CES de Poitiers, CES d’Angoulême, CES de La Roche-sur-Yon, CES de Saint-Nazaire, CES de Cholet, CES d’Angers, CES du Mans, CES de Saint-Brieuc, CES de Rennes, CES de Niort, CES de Dijon, CES de Clermont-Ferrand, CES de Chalon-sur-Saône, CES de Luce (Chartres), CES de Blois, CES de La Riche (Tours), CES de Châteauroux, CES de Saint Doulchard (Bourges), CES de Limoges, CES de Tulle, CES d’Orléans, CES de Bordeaux, CES de Pau, CES de Tarbes, CES de Cahors, CES de Montauban, CES de Auch, CES de Toulouse, CES de Boe (Agen), CES de Rodez, CES de Nîmes, CES de Avignon, CES de Nice, CES de Marseille, CES de Toulon, CES de Frottey-Les-Vesoul (Vesoul), CES de Saint-Etienne, CES d’Annecy, CES de Belfort, CES de Roanne, CES de Lyon, CES de Bourg-en-Bresse, CES de Chambéry, CES de Villefranche-sur-Saône, CES de Saint Martin d'Heres (Grenoble), CES de Reims, CES de Metz, CES de Strasbourg, CES de Sélestat, CES de Colmar, CES de Mulhouse, CES de Saint-Dizier, CES de Vandœuvre-lès-Nancy (Nancy), CES de Longwy

**Laboratories :** Département de Biologie Intégrée (CHU de Grenoble), Inserm U.561 (Hôpital de Saint Vincent de Paul, Paris), Institut Pasteur de Lille ; Laboratoire de BONHOURE de Nemours, Laboratoire de Paris-CPAM, laboratoire de Paris Centre IPC, laboratoire d’analyses médicales Gallimard de Versailles ; Laboratoire de Douai, Laboratoire de HUTTIN-LANOTTE de Dunkerque, Laboratoire BIOPAJ de Le Cateau, Laboratoire de Biologie Spécialisé de l’Institut Pasteur de Lille, Laboratoire du Centre Hospitalier de Roubaix, Laboratoire de Biologie Médicale du Centre Hospitalier de Senlis, Laboratoire du Centre Hospitalier G.ODRON de Tourcoing, Laboratoire BIOPAJ de Valenciennes, Laboratoire de l'Institut inter-Régional pour la Santé (IRSA) de La Riche, Laboratoire d'analyses médicales de l'hôtel de Ville de Sotteville-lès-Rouen ; Laboratoire du CES d'Angoulême, Laboratoire de Biologie Médicale du Centre Hospitalier Départemental de la Roche-sur-Yon, Laboratoire du Centre d'Examens de Santé de Pau, Laboratoire du Centre d'Examens de Santé de la CPAM de la Vienne, Laboratoire du CES de Rennes, Laboratoire du CES de Saint-Nazaire, Laboratoire du CES de Saint-Brieuc ; Laboratoire BIOLAB de Chalon-sur-Saône, Laboratoire du CES de Dijon, Laboratoire Duprat Mignet Roussie de Limoges, Laboratoire de Lucé, Laboratoire du CES d'Orléans, SELAFA - Laboratoire d'Analyses Médicales de Blois, Laboratoire Laporte Molas de Tulle ; Laboratoire du Centre d'Examen de Santé d'Agen Boé, Laboratoire du Centre d'Examen de Santé de Bordeaux, laboratoire du Centre d’Examen de Santé de Pau, Laboratoire du CHU de Toulouse ; Laboratoire DORIA (CESAM 13) du CES de Marseille, Laboratoire CES/CPAM des Alpes-Maritimes, Laboratoire HOYET-ZARANIS de Nîmes, laboratoire d'analyses médicales et de biologie de Narbonne ; Laboratoire d'Analyses Médicales BERGER de Bourg-en-Bresse, Laboratoire d'analyses de Biologie Médicale de Grenoble, Laboratoire du Parc de Lyon, Laboratoire Clinilab de Meylan, Laboratoire du Centre d'Examen de Santé de Mulhouse, Laboratoire de Biologie et de pathologie Médicale de Saint-Chamond ; Laboratoire du CMP de Vandœuvre-lès-Nancy, Laboratoire de l'Avenue de Champagne de Epernay, Laboratoire LABM - Centre de Santé MGEN de Strasbourg.
