# Supplementary material for: Metabolic risk factors in young adults infected with HIV since childhood compared with the general population
Source: PLoS One. 2018 Nov 8;13(11):e0206745. doi: 10.1371/journal.pone.0206745 (PMC6226109; doi:10.1371/journal.pone.0206745)
Supplement: S4 File — (DOC) [file pone.0206745.s004.doc]

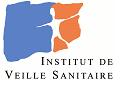

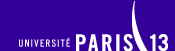
**S4 File. Selection of the ENNS questionnaires, in French and in English**

| Unité de Surveillance et d’Epidémiologie Nutritionnelle (Usen)  *Institut de Veille Sanitaire / Université de Paris 13 / Conservatoire National des Arts et Métiers*  Département des Maladies Chroniques et Traumatismes  Département Santé Environnement  *Institut de Veille Sanitaire* | |
| --- | --- |
| **Etude Nationale Nutrition Santé**  **ENNS**  **2005 – 2006** |  |
|  |  |

**Sélection de l’auto-questionnaire Adultes**

1. Actuellement, prenez-vous des médicaments ?

Pour les femmes : pensez à la pilule contraceptive, au traitement hormonal substitutif, etc.

|  | Oui | 1 |
| --- | --- | --- |
|  | Non | 2 |
|  | Vous ne savez pas | 3 |

1. Indiquez dans le tableau ci-dessous le(s) nom(s), forme(s) de présentation, dosage(s), et nombre(s) de prises par jour du ou des médicaments que vous prenez.

## *Aidez-vous des boites en recopiant ce qui est écrit dessus.*

## *Merci de présenter ces boites lors de la visite de récupération du questionnaire.*

| **Nom du médicament** | Forme de présentation  ***(comprimés, suspension / sirop, suppositoires, injection, patch, etc.)*** | **Dosage**  *Si indiqué sur la boite* | **Nombre de prises**  **par jour** |
| --- | --- | --- | --- |
| *Exemple : Aspirine* | *Comprimé* | *500 mg* | *2 comprimés* |
|  |  |  |  |
|  |  |  |  |
|  |  |  |  |

1. Parmi ces médicaments, y en a-t-il que vous prenez pour diminuer votre tension (ou

| **pression artérielle) ?** | Oui | 1 |
| --- | --- | --- |
|  | Non | 2 |
|  | Vous ne savez pas | 3 |

1. Parmi ces médicaments, y en a-t-il que vous prenez pour diminuer votre cholestérol ?

|  | Oui | 1 |
| --- | --- | --- |
|  | Non | 2 |
|  | Vous ne savez pas | 3 |

1. Parmi ces médicaments, y en a-t-il que vous prenez pour diminuer votre glycémie (sucre

| **dans le sang) parce que vous êtes diabétique ?** | Oui | 1 |
| --- | --- | --- |
|  | Non | 2 |
|  | Vous ne savez pas | 3 |

**Sélection du questionnaire Adultes**

1. **Quelle est votre date de naissance ?**  **/___/___/ / /___/___/ / /___/___/___/___/**

**ou année de naissance /___/___/___/___/ ** *Ne sait pas*

1. **Sexe**

 Homme  Femme

1. Quelle est votre situation matrimoniale actuelle ?

|  | - Célibataire | 1 |
| --- | --- | --- |
|  | - Marié(e) | 2 |
|  | - En couple non marié (PACS, concubinage…) | 3 |
|  | - Veuf(ve) | 4 |
|  | - Divorcé(e) ou séparé(e) | 5 |
|  | - *Refuse de répondre* | 6 |

| Quel est votre pays de naissance ? | - France métropolitaine |
| --- | --- |
|  | - Autre |

1. *Si pays de naissance différent de la France métropolitaine :* préciser le pays ……………………………
2. Quelle est votre situation professionnelle actuelle ?

|  | - Occupe un emploi | 1 |
| --- | --- | --- |
|  | - Chômeur inscrit ou non à l’ANPE | 2 |
|  | - Etudiant, élève, en formation, en stage non rémunéré | 3 |
|  | - Retraité(e) (ancien salarié) ou préretraité(e) | 4 |
|  | - Retiré(e) des affaires (ancien agriculteur, ancien artisan, ancien commerçant…) | 5 |
|  | - Femme ou homme au foyer | 6 |
|  | - Autre inactif (y compris les titulaires d’une pension de réversion ou invalide) | 7 |
|  | - *Refuse de répondre* | 8 |

1. Quel est le diplôme d'enseignement général ou technique le plus élevé que vous ayez obtenu ?

| Montrer la liste. | Aucun, vous n’avez jamais été scolarisé et vous n’avez jamais appris à lire et à écrire | 0 |
| --- | --- | --- |
|  | Aucun, vous n’avez jamais été scolarisé mais vous avez appris à lire et écrire | 1 |
|  | Aucun, votre scolarité s’est arrêtée à l’école primaire | 2 |
|  | Aucun, votre scolarité s’est arrêtée au collège | 3 |
|  | Aucun, votre scolarité s’est arrêtée au-delà du collège | 4 |
|  | Aucun, sans autre précision | 5 |
|  | Certificat d'études primaires (CEP), diplôme de fin d'études obligatoires | 6 |
|  | CAP, BEP, BEPC, brevet élémentaire, BEPS | 7 |
|  | Brevet de technicien, BP (Brevet Professionnel), BEI, BEC, BEA | 8 |
|  | Baccalauréat technologique ou professionnel | 9 |
|  | Baccalauréat général | 10 |
|  | BTS, DUT, DEST, DEUG (y compris formation paramédicale ou sociale) | 11 |
|  | 2e ou 3e cycle universitaire, grande école | 12 |
|  | Autre (préciser) : | 13 |
|  | *Refuse de répondre* | 14 |

1. Est-ce que vous fumez, même de temps en temps ?

|  | Oui, vous fumez quotidiennement | 1 |
| --- | --- | --- |
|  | Oui, vous fumez occasionnellement (moins d’une cig./j) | 2 |
|  | Non, vous ne fumez pas et vous avez déjà fumé | 3 |
|  | Non, vous ne fumez pas et vous n’avez jamais fumé | 4 |
|  | *Refus* | 5 |

**Sélection du questionnaire « Volet clinico-biologique »**

7. *Si le sujet est une femme*

Etes-vous enceinte ? ❒ Oui ❒ Non ❒ Ne sait pas

16. Anthropométrie Poids : | _ | _ | _ |,| _ | kg Taille : | _ | _ | _ | ,| _ | cm

Tour de taille : | _ | _ | _ | cm Tour de hanches : | _ | _ | _ | cm

17. Pression artérielle

systolique : | _ | _ | _ | mm d’Hg diastolique : | _ | _ | _ | mm d’Hg

Les variables biologiques ont été directement envoyées par les laboratoires biologiques au centre de gestion des données.
