# Supplementary material for: Metabolic risk factors in young adults infected with HIV since childhood compared with the general population
Source: PLoS One. 2018 Nov 8;13(11):e0206745. doi: 10.1371/journal.pone.0206745 (PMC6226109; doi:10.1371/journal.pone.0206745)
Supplement: S1 Table — (DOCX) [file pone.0206745.s006.docx]

**S1 Table: Sensitivity analysis: comparison of prevalence of metabolic abnormalities of patients infected with HIV since childhood with the general population born in France (COVERTE and ENNS), adjusted for being overweight and education level, by logistic regression.**

|  | **MEN** | | |  | **WOMEN** |  |
| --- | --- | --- | --- | --- | --- | --- |
|  | aOR* | **95%CI** | **p-value** | aOR* | **95%CI** | **p-value** |
| Overweight | 0.5 | 0.2-1.2 | 0.124 | 1.0 | 0.5-2.1 | 0.936 |
| Elevated blood pressure | 1.5 | 0.6-3.5 | 0.332 | 1.6 | 0.6-3.9 | 0.328 |
| **Elevated waist circumference** | 1.4 | 0.3-6.7 | 0.642 | **10.5** | **3.3-33.0** | **<10^-4^** |
| Elevated fasting glucose | 0.9 | 0.3-2.7 | 0.879 | ND | ND | ND |
| **Elevated triglycerides** | **7.0** | **2.4-19.9** | **0.003** | 1.1 | 0.3-3.6 | 0.921 |
| **Reduced HDL-cholesterol** | **2.9** | **1.1-7.3** | **0.022** | **2.4** | **1.2-4.7** | **0.013** |
| Elevated LDL-cholesterol | 1.3 | 0.5-3.1 | 0.605 | 0.8 | 0.3-1.9 | 0.636 |
| **Elevated total cholesterol** | 1.7 | 0.8-3.7 | 0.202 | **0.5** | **0.2-1.0** | **0.058** |
| **Metabolic syndrome** | 4.7 | 1.0-23.0 | 0.055 | **9.6** | **1.5-59.8** | **0.015** |

aOR: adjusted Odds Ratio; CI: confidence interval; HDL: high-density lipoprotein; LDL: low-density

**^*^** using logistic regression adjusted for educational level and being overweight (except for overweight adjusted on educational level only), taking into account sampling weights
